# Nurses’ knowledge regarding recommended practices on using surgical attire in operating theatre

**DOI:** 10.4102/hsag.v29i0.2469

**Published:** 2024-02-29

**Authors:** Joshua Alayemi, Wilma ten Ham-Baloyi, Sihaam Jardien-Baboo

**Affiliations:** 1 Department of Nursing Science, Faculty of Health Sciences, Nelson Mandela University, North Campus, Gqeberha, South Africa

**Keywords:** continuing nursing education, surgical attire, surgical wound, cross infection, nurses, operating theatre, knowledge

## Abstract

**Background:**

To reduce the risk for surgical site infections, nurses in the operating theatre environment must have knowledge of and adhere to recommended practices regarding the use of surgical attire.

**Aim:**

To evaluate the effect of an educational intervention on nurses’ knowledge related to recommended practices regarding the use of surgical attire in operating theatre.

**Setting:**

Operating theatres in two public and two private hospitals in the Eastern Cape province, South Africa.

**Methods:**

An educational pilot study, using a quasi-experimental, two-group pre- and post-test design, was conducted. A convenience sample of *n* = 85 nurses was purposively allocated to a control group and an intervention group. An existing educational intervention consisting of an interactive training session, brochures based on the Association of periOperative Registered Nurses’ (AORN) guidelines and a summary of these guidelines was implemented for the intervention group, while the control group received only the summary of the guidelines. Data were collected through self-administered pre- and post-test questionnaires from March 2019 to August 2019.

**Results:**

The overall knowledge score for nurses in the intervention group post-intervention improved with a large significance (*p* ≤ 0.000 and Cohen’s d = 1.26).

**Conclusion:**

The intervention has shown potential to improve the knowledge related to recommended practices of nurses in operating theatres regarding the use of surgical attire.

**Contribution:**

This pilot study encourages the implementation of the intervention on the use of surgical attire but requires further development and a wider implementation to measure its impact, and access to resources, enhancing and sustaining its success.

## Introduction

The operating theatre presents a demanding and dynamic setting for nurses, where they utilise cutting-edge equipment and technologies to assist the surgeon conducting surgical procedures (Blomberg, Bisholt & Lindwall 2018). Failure to adhere to infection control and prevention in this environment can lead to patients developing surgical site infections and sepsis (Al Laham 2012). Surgical site infections manifest following the completion of a surgical procedure and represent the most prevalent complication that occurs in patients after surgery (Russo, Watkins & Centers for Disease Control and Prevention 2018). It is estimated that 8% of hospital-acquired infections are categorised as surgical site infections, and an estimated 2% – 5% of patients undergoing surgery may experience surgical site infections during their hospitalisation, potentially resulting in prolonged hospital stays of patients as well as more hospital costs and an increased risk for long-term infections or even death of patients (Mockford & O’Grady 2017; O’Brien, Gupta & Itani 2020; Safe Care Campaign 2018).

Enhancing the prevention of surgical site infections can be achieved by maintaining proper handling of surgical equipment, ensuring a clean and sterile environment and utilising the appropriate and correct surgical attire throughout surgical procedures (Jenkins 2017). There is evidence suggesting that clean and sterile surgical attire, including gloves and surgical gowns (to a large extent), as well as skull caps, a surgical mask, scrubs, boots and an apron (to a lesser extent), can prevent the transmission of microorganisms from healthcare personnel (including nurses) to the patient, reducing the risk for surgical site infections in this setting (Association of periOperative Registered Nurses’ [AORN] 2018; Cowperthwaite & Holm 2015; McHugh et al. 2014; Salassa & Swiontkowski 2014). For example, surgical attire, such as surgical masks, if correctly worn, covering the mouth and nose, safeguard patients from pathogens originating within the surgical team. Further, wearing of clean, dry sterilised surgical scrubs and gowns as well as headgear covering the entire head reduce the outflow or shedding of organisms from the surgical team to the patient, which decreases the number of bacteria in the air (Cowperthwaite & Holm 2015; McHugh et al. 2014). Wearing of dedicated shoes or boots that are used only in operating theatre or shoe covers and cleaning of handheld mobile devices such as cell phones prevent contamination of the operative field (Tateiwa et al. 2020). Further, home laundering of surgical scrubs is not recommended as it lacks required parameters for hot and/or cold water temperature, detergent or water treatments (AORN 2015).

To reduce the risk for surgical site infections in the operating theatre environment, it is therefore important for nurses to have knowledge of and adhere to recommended practices regarding the use of surgical attire (AORN 2016). Ongoing education and training through, for example, educational interventions could assist in promoting nurses’ knowledge on recommended practices in the clinical field, including the use of surgical attire in operating theatres. Educational interventions have been repeatedly used to improve nurses’ knowledge regarding recommended practices, with promising results (Häggman-Laitila, Mattila & Melender 2016). Although few educational interventions have been implemented among nurses in the operating theatre (Fereidouni et al. 2022; Kalantari et al. 2021; Sutherland-Fraser et al. 2012), no research has been published in South Africa regarding an educational intervention that influences nurses’ knowledge on recommended practices related to the use of surgical attire. Therefore, this educational pilot study aimed to evaluate the effect of an educational intervention on nurses’ knowledge regarding recommended practices related to the use of surgical attire in operating theatre.

## Research methods and design

### Study design

An educational pilot study, employing a quasi-experimental, two-group, pre-post- test design, was conducted from March 2019 to August 2019. Participants were selected to an intervention group or a control group. Both groups received a pre- and a post-test questionnaire. The primary outcome of the study was to improve nurses’ knowledge regarding recommended practices related to the use of surgical attire in operating theatre. The secondary outcome (although not measured in this study) included reduced numbers of surgical site infections, length of stay and patient mortality.

### Setting

The study was conducted in one of the six health districts in the Eastern Cape Province, South Africa. The four largest hospitals in the health district – two private and two public hospitals – were included in the study. Each hospital has a cardiac and/or thoracic theatre, urology theatre, orthopaedic theatre, ophthalmology theatre, neurological theatre and general theatre where laparoscopic and diagnostic interventions and surgical operations are done. Nurses working in these operating theatres are all trained (whether formal through postgraduate qualifications in Operating Theatre Nursing or informal through in-service training) to offer care and perform scrub, anaesthetic, circulating and recovery functions. Most nurses in the study setting rotate in these functions while a few have specialised in these functions and do not rotate. At the time of the study, institutional statistics in some of the hospitals included in the study revealed surgical site infections to be as high as 10.0% (Opadotun 2019). The researcher noted a deficiency in knowledge among professional nurses working in operating theatres, which led to practices that heightened the likelihood of surgical site infections. These practices included the reuse of surgical gowns upon returning to the operating theatre, inadequately covering the hair with head covers, improper hand hygiene, such as not thoroughly drying hands up to the elbow, resulting in water dripping onto surgical scrubs and gowns before commencing scheduled operations (Jenkins 2017; Mockford & O’Grady 2017).

### Participants

Because of the small sample size of the population and the geographic proximity of the selected hospitals, which could have led to possible contamination of the intervention, randomisation was not possible. Therefore, convenience sampling was used to select all 85 nurses, working at operating theatres, offering care and performing functions related to scrub, anaesthetic, circulating and/or recovery in four hospitals, which were purposively allocated to an intervention group (Hospital 3 – public and Hospital 4 – private) and a control group (Hospital 1 – public and Hospital 2 – private). To have similar sample sizes, one private hospital and one public hospital were purposively selected to be included in each group.

**FIGURE 1:**
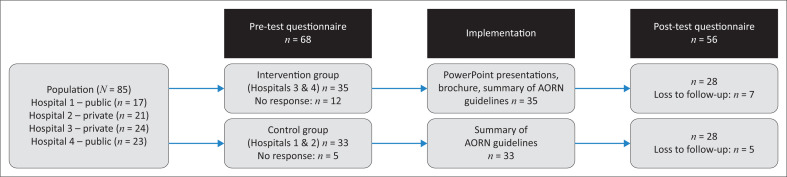
Sampling framework per group.

Because of the limited population size, there was no need to compute a minimum sample size, and the research plan aimed to include the largest possible sample size. In total, 68 nurses participated in the pre-test questionnaire, and out of these participants, 56 were involved in the post-test questionnaire. The sampling framework is illustrated in Figure 1.

### The educational intervention

An existing educational intervention, based on the AORN (2015) guidelines regarding the use of surgical attire in the prevention of surgical site infections in operating theatres, was used. The AORN guidelines were chosen because it covers all the topics related to the use of surgical attire – namely headgear, surgical facemasks, laundering of surgical scrubs, shoe covers, surgical scrub suits, surgical gown, jewellery, gloving, personal protective equipment (PPE) (eye gear and aprons) and electronic devices (e.g., cell phones, tablets and other personal handheld devices). Furthermore, these are evidence-based guidelines that specifically emphasise patient safety and the achievement of optimal outcomes, and they have been previously put into practice (AORN 2015).

The educational intervention consisted of an interactive training session, brochures, as well as a summary of the AORN guidelines. The educational intervention was deemed suitable as the methods were not time consuming and could be implemented in a busy environment, such as the operating theatre, without disruption of patient care (see Table 1).

**TABLE 1:** Contents of the educational intervention for the intervention and control groups.

Educational tool	Mode of delivery	Content AORN guidelines (AORN 2015)
**Intervention group**		
27 Slides PowerPoint presentation based on original presentation by Seifert et al. on behalf of AORN (2015)	20-min, face-to-face, interactive training session	One slide listing the objectives of the session. Pictures and text, outlining the reasons, recommended use and justification of the following: ■ Head covering ■ Surgical face mask ■ Surgical scrub suits ■ Shoe covers ■ Home laundering of surgical attire ■ Jewellery ■ Gloving ■ PPE: eyewear and aprons ■ Electronic devices (cell phones, tablets and other personal handheld devices)
Four-page brochure	Hardcopy, distributed to participants immediately after the presentation as part of the training session	Pictures and text, outlining the recommended use and justification of the following: Headgear Surgical face mask Scrub suits Shoe covers Home laundering of surgical attire Jewellery Gloving PPE: eyewear and aprons Electronic devices (cell phones, tablets and other personal and handheld devices)
**Intervention and control groups**		
Two-page summary of the AORN guidelines (AORN 2015)	Hardcopy, left at each nursing station of the operating theatre in the selected hospitals	Text: Takeaway message and explanation of surgical attire (laundering and type of scrub attire), covering of arms, jewellery, and electronic equipment (cell phones, tablets and other personal handheld devices)

AORN, Association of periOperative Registered Nurses; PPE, personal protective equipment.

For the intervention group, a once-off 20-min interactive training session was conducted with all participating nurses that offer care and perform functions related to scrub, anaesthetic, circulating and recovery on day duty by the first author, a male registered nurse. The intervention was conducted consistently for both of the two hospitals in the intervention group. It occurred at the same time of day during a week typically considered to have lower activity, with fewer scheduled operations. A private, quiet room was used to avoid participant fatigue and enhance their attention. On the same day, for all participating night staff working 19:00–07:00, the training session was repeated at 19:30 after handover. The session was followed by the distribution of hardcopy brochures to each participating nurse, and a hardcopy of a summary of the AORN guidelines was left at each nursing station of the operating theatre in the selected hospitals. The brochures and the summary of the guidelines aimed at reiterating the information in the training session and permitted nurses to become acquainted with the suggested evidence-based practices related to the utilisation of surgical attire in preventing surgical site infections.

In the control group, participants in the designated two hospitals received only a summary of the AORN guidelines, which was ethically required because of high surgical site infection rates across selected hospitals in both groups. The implementation timeframe was 3 months, which was deemed acceptable to provide sufficient time for participants to familiarise themselves with the AORN guidelines and recommended practices regarding the use of surgical attire, as recommended elsewhere in a similar setting (Falconer et al. 2021). Nurses could contact the first author telephonically or via email if they had any concerns or questions during the implementation timeframe.

A review panel consisting of two individuals holding honours degree in Advanced Nursing in Operating Theatre and one with a master’s degree in Operating Theatre Nursing reviewed the educational intervention. Each of the reviewers possessed significant experience, ranging from 10 to 20 years, in both public and private operating theatre care. This collective experience was instrumental in evaluating the intervention’s feasibility, appropriateness for the South African context and its overall design and readability prior to its implementation.

### Pre- and post-test questionnaires

Data were collected by means of hardcopy self-administered pre- and post-test questionnaires. The pre-test questionnaire was the same as the post-test questionnaire, and the questionnaires were the same for both groups in order to test the nurses’ knowledge regarding recommended practices related to the use of surgical attire. The pre- and post-test questionnaires were formulated and designed in alignment with the existing educational intervention based on the AORN guidelines regarding the use of surgical attire (AORN 2015; Braswell & Spruce 2012). The questionnaires included two sections – section one entailed demographic questions (4 items, involving participants’ basic and additional education, working experience and affiliated hospital) and section two consisted of a total of 31 items related to 11 practices on the use of surgical attire to prevent surgical site infections in operating theatres, as follows: guideline adherence, headgear, surgical facemask, laundering of surgical scrubs, shoe covers, scrub suits, surgical gown, gloving, jewellery, PPE: eye gear and aprons, and electronic devices. Possible responses for each item included ‘Yes’, ‘No’ or ‘Don’t know’. To enhance validity, the questionnaires were reviewed by the same three reviewers who also reviewed the educational intervention. No amendments to the questionnaires were required.

### Data collection

After the relevant ethical clearance and permissions from each hospital were obtained, all nurses at the selected operating theatres were approached during their tea breaks, provided with both verbal and written information about the study and invited to participate in the study. Those who agreed to participate in the study completed consent forms that were issued prior to completion of the questionnaire. The first author distributed the questionnaires to the participants for completion. Participants were asked not to discuss items or answers among each other or use any information to answer the questionnaire. The completed questionnaires were collected by the first author immediately after completion. The questionnaire took approximately 15 min to complete. No incentives were given to participants to partake in the study.

Data for the post-test were collected in the same manner as the pre-test questionnaire. For both groups, the same pre- and post-questionnaires were used. This was done after the 3-month post-implementation phase of the intervention. After the post-test was completed, the full educational intervention was shared with the nurses in the control group.

### Data analysis

The pre-test and/or post-test data were captured and analysed employing descriptive and inferential statistics in Microsoft Excel, with the assistance of a statistician. The chi-square test of independent variance was used to determine any significant relationships between the responses. A chi-square *p*-value less than 0.05 was considered statistically significant. Cohen’s d was used to determine whether the improvement in knowledge (post- minus pre-test scores) was significant, with values 0.2 (small), 0.5 (medium) and 0.8 (large). The knowledge regarding recommended practices’ scores of 1.00–3.99 was regarded low, while 4.00–4.38 was regarded average and 4.39–5.00 was regarded high.

### Ethical considerations

The study received approval from the Institutional Review Board of Nelson Mandela University, under Approval Number H18-HEA-NUR-020. Additionally, consent was sought from all participants, and their involvement in the study was entirely voluntary. The questionnaires used were anonymous and did not seek any personally identifiable information.

## Results

Out of a total of 85 potential participants, 68 participated in the pre-test (33 from the control group and 35 from the intervention group), resulting in an overall 80.0% response for the pre-test. A total of 56 participants participated in the post-test (28 in the control group and 28 in the intervention group), resulting in an overall 65.1% response rate for the post-test.

### Demographic data

The demographic data are outlined in Table 2. Demographic data were similar for both the control and intervention groups. The main findings for both groups are therefore as follows: most participants had a bridging course (aimed at upskilling nurses without a diploma in nursing to a 3-year diploma containing basic training as a nurse and midwife) or a 4-year diploma (basic training as a nurse and midwife without honours or specialised training in operating theatre nursing) and about half had an additional qualification (honours or master’s degree in Operating Theatre Nursing). Most participants had more than 5 years of work experience. A majority of the participants in the control group and a slightly smaller portion in the intervention group were employed in private hospitals. No statistical significance was found for the demographic characteristics for both pre- and post-test.

**TABLE 2:** Demographic data.

Demographic items	Control group (*N* = 61)				Intervention group (*N* = 63)				*p* pre-test	*p* post-test
	Pre-test (*n* = 33)		Post-test (*n* = 28)		Pre-test (*n* = 35)		Post-test (*n* = 28)			
	*n*	%	*n*	%	*n*	%	*n*	%		
**Basic qualification**	11	33.3	9	32.2	13	37.1	10	35.7	0.167	0.904
4-year diploma	17	51.5	13	46.4	10	28.6	11	39.3	-	-
Bridging course	4	12.1	6	21.4	8	22.9	7	25.0	-	-
4-year degree	1	3.1	0	0.0	4	11.4	0	0.0	-	-
**Additional qualification**										
Yes	14	42.4	14	50.0	17	48.6	15	53.6	0.514	0.789
No	19	57.6	14	50.0	18	51.4	13	46.4	-	-
**Years of working experience**										
Less than a year	9	27.2	3	10.7	3	8.6	2	7.1	0.213	0.594
1–2 years	5	15.2	5	17.9	7	20.0	4	14.3	-	-
3–4 years	4	12.1	5	17.9	3	8.6	2	7.1	-	-
5–9 years	5	15.2	7	25.0	4	11.4	7	25.0	-	-
10 and above	10	30.3	8	28.5	18	51.4	13	46.5	-	-
**Private or public hospital**										
Private	19	57.6	17	60.7	17	48.6	12	42.9	0.457	0.181
Public	14	42.4	11	39.3	18	51.4	16	57.1	-	-

### Knowledge regarding recommended practices related to the use of surgical attire

The t-test scores for knowledge regarding recommended practices related to the use of surgical attire are outlined in Table 3.

**TABLE 3:** T-test scores for knowledge on recommended practices regarding the use of surgical attire for control groups and intervention groups pre- and post-test.

Variables	Group	Pre- and/or post-test	*n*	Mean	SD	Difference	t	*d.f.*	*p*	Cohen’s d
Score 1 (guidelines)	Control	Pre-test	33	4.73	0.67	0.98	5.53	59	**< 0.000**	**1.38 Large**
		Post-test	28	3.75	0.75	-	-	-	-	-
	Intervention	Pre-test	35	4.63	0.65	0.24	1.31	61	0.196	-
		Post-test	28	4.39	0.79	-	-	-	-	-
Score 2 (headgear)	Control	Pre-test	33	3.25	1.05	−0.18	−0.71	59	0.478	-
		Post-test	28	3.43	0.89	-	-	-	-	-
	Intervention	Pre-test	35	2.48	0.83	−1.20	−2.02	60	**< 0.000**	**1.24 Large**
		Post-test	28	3.68	1.13	-	-	-	-	-
Score 3 (facemask)	Control	Pre-test	33	3.84	0.42	−0.14	−1.44	59	0.154	-
		Post-test	28	3.98	0.30	-	-	-	-	-
	Intervention	Pre-test	34	3.77	0.65	−0.28	−2.02	60	**0.048**	**0.52 Medium**
		Post-test	28	4.07	0.35	-	-	-	-	-
Score 4 (laundering of surgical scrubs)	Control	Pre-test	33	4.21	1.34	0.43	1.18	59	0.242	-
		Post-test	28	3.79	1.47	-	-	-	-	-
	Intervention	Pre-test	35	3.17	1.82	−1.01	−2.56	61	**0.013**	**0.65 Medium**
		Post-test	28	4.18	1.12	-	-	-	-	-
Score 5 (shoe covers)	Control	Pre-test	33	4.54	0.64	0.35	1.83	59	0.073	-
		Post-test	28	4.19	0.83	-	-	-	-	-
	Intervention	Pre-test	35	4.09	0.97	−0.49	−2.49	61	**0.019**	**0.61 Medium**
		Post-test	28	4.58	0.50	-	-	-	-	-
Score 6 (scrub suits)	Control	Pre-test	33	3.71	0.71	−0.03	−0.22	59	0.827	-
		Post-test	28	3.74	0.41	-	-	-	-	-
	Intervention	Pre-test	35	3.42	0.74	−0.32	−2.06	61	**0.044**	**0.52 Medium**
		Post-test	28	3.74	0.39	-	-	-	-	-
Score 7 (surgical gown)	Control	Pre-test	33	4.67	0.58	−0.04	−0.26	59	0.798	-
		Post-test	28	4.70	0.49	-	-	-	-	-
	Intervention	Pre-test	35	4.62	0.66	−0.19	−1.33	61	0.189	-
		Post-test	28	4.81	0.41	-	-	-	-	-
Score 8 (jewellery)	Control	Pre-test	22	4.49	1.09	0.46	1.38	43	0.175	-
		Post-test	23	4.03	1.13	-	-	-	-	-
	Intervention	Pre-test	27	4.21	1.11	−0.34	−1.23	46	0.223	-
		Post-test	21	4.56	0.73	-	-	-	-	-
Score 9 (gloving)	Control	Pre-test	33	4.70	0.43	−0.07	−0.66	59	0.510	-
		Post-test	28	4.77	0.40	-	-	-	-	-
	Intervention	Pre-test	35	4.27	0.68	−0.21	−1.19	61	0.240	-
		Post-test	28	4.48	0.73	-	-	-	-	-
Score 10 (personal protective equipment: eye gear and aprons)	Control	Pre-test	32	3.04	1.16	−0.63	−2.41	58	**0.019**	**0.62 Medium**
		Post-test	28	3.67	0.77	-	-	-	-	-
	Intervention	Pre-test	33	3.39	1.12	−0.30	−1.22	59	0.229	-
		Post-test	28	3.70	0.75	-	-	-	-	-
Score 11 (electronic devices, e.g. cell phones, tablets and other personal handheld devices)	Control	Pre-test	32	2.47	1.54	−1.71	−4.84	58	**< 0.000**	**1.25 Large**
		Post-test	28	4.18	1.12	-	-	-	-	-
	Intervention	Pre-test	33	2.39	1.34	−1.86	−5.36	59	**< 0.000**	**1.38 Large**
		Post-test	28	4.25	1.35	-	-	-	-	-
Overall score	Control	Pre-test	33	3.96	0.40	−0.06	−0.57	59	0.572	-
		Post-test	28	4.01	0.38	-	-	-	-	-
	Intervention	Pre-test	35	3.68	0.47	−0.53	−4.97	61	**< 0.000**	**1.26 Large**
		Post-test	28	4.22	0.36	-	-	-	-	-

Note: Chi-square: *p*-value less than 0.05 is statistically significant (in bold); Cohen’s d 0.2 (small), 0.5 (medium) and 0.8 (large) significant (in bold).

*n*, natural numbers; SD, standard deviation; t, time; *d.f.*, degrees of freedom.

Out of the 11 knowledge items related to recommended practices for the use of surgical attire to prevent surgical site infections, the control group showed a substantial and statistically significant improvement in their understanding of the guidelines for surgical attire to prevent surgical site infections (Score 1) (*p* < 0.000 and Cohen’s d = 1.38). Additionally, a substantial and statistically significant improvement was observed in their understanding regarding the cleaning of electronic devices when entering and leaving the operating theatre (Score 11) (*p* < 0.000 and Cohen’s d = 1.25) for the control group. A moderate and statistically significant difference was also noted in knowledge regarding the use of personal protective equipment in the theatre (Score 10) (*p* = 0.019 and Cohen’s d = 0.62).

In the intervention group, a significant and substantial improvement was observed between the pre-test and post-test questionnaires for participants’ knowledge on the use of headgear in the operating theatre (Score 2) by the participating nurses (*p* < 0.000 and Cohen’s d = 1.24). Similarly, a substantial and statistically significant improvement was noted in knowledge for the cleaning of electronic devices (Score 11) (*p* < 0.000 and Cohen’s d = 1.38) in the same group. Moderate significance was found in participants’ knowledge regarding the use of surgical facemasks to prevent surgical site infections (Score 3) (*p* = 0.48 and Cohen’s d = 0.52), as well as in the laundering of surgical scrubs (Score 4) (*p* = 0.013 and Cohen’s d = 0.65), shoe cover practices (Score 5) (*p* = 0.019 and Cohen’s d = 0.61) and the use of scrub suits in the operating theatre (Score 6) (*p* = 0.044 and Cohen’s d = 0.52).

Knowledge scores of 1.00–3.99 were regarded low, while 4.00–4.38 were regarded average and 4.39–5.00 were regarded high. The nurses’ knowledge scores pertaining to recommended practices were generally low for both the control and intervention groups during the pre-test, and they reached an average level in the post-test. Notably, there was a significant and substantial improvement in the intervention group between the pre-test and post-test questionnaires (*p* < 0.000 and Cohen’s d = 1.26).

## Discussion

This pilot study aimed to evaluate the effect of an educational intervention on nurses’ knowledge regarding recommended practices related to the use of surgical attire in operating theatre. The findings of the study indicated that the educational intervention, which included a 20-min interactive training session, distribution of brochures and a summary of the AORN 2015 guidelines related to the use of surgical attire, had a beneficial impact. Specifically, it improved the nurses’ understanding of the recommended practices for using surgical attire to prevent surgical site infections in operating theatres, aligning with AORN’s recommendations. This finding was congruent with other, similar research studies (Bassyouni, Wegdan & El-Sherbiny 2016; Rinaldi et al. 2016). Hence, it is advisable to put this educational intervention into practice within operating theatres. However, when implementing the educational intervention, the latest AORN 2020 guidelines on the use of surgical attire should be used (Link 2020), as this version was not yet published when the pilot study was conducted.

In this study, knowledge regarding certain practices related to the AORN 2015 guidelines regarding the use of surgical attire, including headgear, face masks, laundering of surgical scrubs, shoe covers, scrub suits and PPE (eye gear and aprons), significantly improved in the post-test in either the control group or the intervention group, while the use of electronic devices improved significantly for both groups. This could be explained as the control group did receive a summary of AORN’s guidelines related to the use of surgical attire, indicating some operating theatre nurses in the control group may have read these guidelines. Information on recommended practices specifically related to the utilisation of head covering in the operating theatre and cleaning of electronic devices should be accessible to all nurses in operating theatre, as these showed a large significant improvement in this study. Although there seems to be a paucity in published intervention studies conducted on the topic, a quantitative study conducted in Egypt as well as an observational study in Turkey found that compliance to correct use of surgical attire and PPE was generally high among nurses in operating theatre (Awadalla, Garas & Hanafy 2019; Gülşen et al. 2021). Furthermore, the pre- and post-test questionnaires conducted in both groups may have led to more awareness of the use of surgical attire among participants.

With regard to demographics, it must be noted that about half of the nurses in this study did not possess an additional post-basic qualification. Possessing an additional education was not found significant related to improved knowledge regarding recommended practices related to the use of surgical attire among our study participants. However, an additional education (such as postgraduate studies, including honours and master’s degree in Operating Theatre Nursing) can lead to an improvement in knowledge reflecting in improved adherence to recommended practices, including the use of surgical attire (McHugh & Lake 2010; Sethi et al. 2018), which is important as knowledge scores among study participants were low (pre-test) and average (post-test) as highest scores. Nurses are thus encouraged to acquire additional qualifications or in-service training (compulsory or optional professional development activities to maintain or upgrade professional qualifications and competencies) as these will help in improving skills and knowledge necessary to meet the complex healthcare demands (University of Texas Arlington 2019). In light of the results from the present study, it is advisable to consider education and training, either formally (such as pursuing an additional qualification) or informally (through regular in-service training) as nurses’ knowledge regarding recommended practices on the use of surgical attire improved post-implementation of the educational intervention irrespective of nurses possessing basic versus specialised training. Furthermore, the questionnaire designed for this study could be used by managers in operating theatres to identify gaps in knowledge regarding recommended practices related to the use of surgical attire in operating theatres, to tailor in-service training to the identified practice gaps and monitor improvement in both knowledge and adherence to recommended practices.

However, to prevent surgical site infections, providing education using an educational intervention that promotes the correct use of surgical attire alone may not be sufficient. It is recommended that the use of surgical attire must be adhered to, together with general infection prevention and control principles, such as hand hygiene and overall cleanliness of the operating theatre, as well as the administration of prophylactic antibiotics and appropriate hair removal procedures without a razor, ensuring normothermia, employing chlorhexidine gluconate in combination with alcohol-based skin preparation agents, conducting decolonisation with intranasal antistaphylococcal agents and antistaphylococcal skin antiseptics for high-risk procedures, monitoring perioperative glucose levels and utilising negative pressure wound therapy have demonstrated effectiveness in diminishing the incidence of surgical site infections (Mckenna, Hutchinson & Butler 2019; Seidelman, Mantyh & Anderson 2023; Woodruff & Hohler 2018). Further, as implementation of strict operating theatre attire policies, including complete covering of both the ears and facial hair, has no proven effect on surgical site infection rates (Farach et al. 2018), consideration should be given to implementing surgical attire in accordance with procedures that are proven to reduce infection rates, especially for countries with limited available resources (Bhutta 2019; Petrilli et al. 2018). Education and training on the use of surgical attire to reduce surgical site infections should therefore include the above infection control principles and interventions while considering the available resources. Subsequently, to facilitate training, a dedicated person with expertise in infection control or an entire multidisciplinary infection control team, consisting of nurses, microbiologists and physicians who have undergone training on infection control, should be appointed as this has proven to enhance knowledge and adherence to infection control practices, including the correct use of surgical attire (Cima et al. 2013).

The research study had the following identified limitations: the findings were derived from data gathered via a self-administered questionnaire, which may not accurately represent the participants’ actual knowledge of recommended practices concerning the use of surgical attire in operating theatres. Consequently, it is advisable to conduct an observational study. Additionally, a reduced level of participation from nurses at certain sites, possibly due to lack of enthusiasm, led to a lower response rate, especially in the post-test questionnaire. The questionnaire did not include age and gender as part of the demographic items and did not measure its reliability and validity. There is thus a need to further develop and test the questionnaire.

Given the time limitations and the study’s restricted focus, the inclusion of healthcare professionals beyond nurses offering care and performing functions related to scrub, anaesthetic, circulating and recovery in operating theatres was not feasible. Hence, findings cannot be generalised to a larger population of nurses in operating theatres and other non-nursing members of the surgical team. Additionally, the educational intervention was based on existing guidelines for the use of surgical attire and included only practices outlined in these guidelines, which may not have included all practices nurses should have knowledge about related to the use of surgical attire. Finally, the current study neither measured the effect of the implemented educational intervention on patient outcomes, including surgical site infection rates, nor its prolonged impact on practice outcomes over time (for example, by repeating the post-test). It is therefore recommended that further development and testing of the educational intervention should be done by conducting a similar study, which includes a larger sample size as well as various members of the multidisciplinary team in the operating theatre, testing its long-term effect on knowledge retention as well as its effects on surgical site infections in the operating theatre setting.

## Conclusion

This pilot study aimed to evaluate the effect of an educational intervention on nurses’ knowledge regarding recommended practices related to the use of surgical attire in operating theatre. The tailored educational intervention, carried out in the two chosen hospitals within the intervention group, demonstrated a significant enhancement in nurses’ knowledge of recommended practices related to the use of surgical attire, particularly the use of headgear in the operating theatre and cleaning of electronic devices, in the prevention of surgical site infections in operating theatres but requires further exploration and testing. For the control group, there were also significant changes in knowledge reported which were not because of the educational intervention. Further, access to evidence-based educational materials, including best practice guidelines, formal education, consideration of general infection control principles and resources, and a multidisciplinary approach would enhance and sustain the success of the intervention.

The findings of this pilot study can be utilised by nurses to enhance knowledge of and adherence to best practices regarding the use of surgical attire to prevent surgical site infections in operating theatres.
